# A new species of *Polyonyx* (Crustacea, Anomura, Porcellanidae) inhabiting polychaete-worm tubes (Annelida, Chaetopteridae) in the Indo-West Pacific

**DOI:** 10.3897/zookeys.818.30587

**Published:** 2019-01-17

**Authors:** Bernd Werding, Alexandra Hiller

**Affiliations:** 1 Institut für Tierökologie und Spezielle Zoologie der Justus-Liebig-Universität Giessen, Heinrich-Buff-Ring 29 (Tierhaus), D-35392 Giessen, Germany Justus-Liebig-Universität Giessen Germany; 2 Smithsonian Tropical Research Institute, Apartado 0843-03092, Panamá, República de Panamá Smithsonian Tropical Research Institute Panama Panama

**Keywords:** adaptation, *
Chaetopterus
*, commensalism, polychaete-inhabiting

## Abstract

*Polyonyxsocialis***sp. n.** from the South China Sea of Vietnam is described. The new species was collected in a previous study that compared the vertebrate and invertebrate symbiont communities living in the tubes of two syntopic species of the polychaete genus *Chaetopterus*. *Polyonyxsocialis***sp. n.** inhabits the tubes of the smaller polychaete species as a heterosexual pair, and frequently shares the cavity of the host’s tube with a larger porcellanid, *P.heok*, also present as a male-female pair, and with a species of trinchesiid nudibranch. Less frequently, the new species shares its host with a heterosexual pair of a larger species of pinnotherid crab. *Polyonyxsocialis***sp. n.** belongs to the *P.sinensis* group, a world-wide distributed morphological line within the heterogeneous genus *Polyonyx*. Most species in this group are obligate commensals of chaetopterid polychaetes. The crabs have a transversally cylindrical habitus, which enables them to move laterally along the worm tubes with ease. *Polyonyxsocialis***sp. n.** is a relatively small species that lives attached to the inner walls of the polychaete tube. The small size and flattened chelipeds and walking legs of the new species confers it an advantage to cohabiting the same worm tube with larger decapod species occupying most of the tube’s cavity.

## Introduction

The porcellanid genus *Polyonyx* Stimpson is a diverse and heterogeneous taxon containing more than 30 species worldwide, most of which distributed in the Indo-West Pacific (IWP) (Johnson 1958; Haig 1960; Werding 2001; Osawa 2007; Osawa and McLaughlin 2010; Osawa and Ng 2016; Osawa et al. 2018; this study). Many species of this genus are known to live commensally with polychaete worms (Haig 1960, 1979; Ng and Sasekumar 1993; Werding 2001; Osawa et al. 2018; own observations). Johnson (1958) arranged the IWP species into three morphological groups, designated as *Polyonyxbiunguiculatus* (Dana 1852), *P.denticulatus* Paul’son, 1875, and *P.sinensis* Stimpson, 1858. Nakasone and Miyake (1969) considered the *P.denticulatus* group as the new genus *Aliaporcellana*, which was later redefined by Haig (1978).

The largest of Johnson’s (1958) assemblages is the *Polyonyxsinensis* group, which is worldwide distributed, though most of the species in this group have an Indo-West Pacific (IWP) distribution. According to this author this group contains species with a “pronounced tendency towards commensalism”. Indeed, most species in this morphological line are commensal (Haig 1964), and have been reported to inhabit the tubes of tube-dwelling polychaetes, mainly of the family Chaetopteridae Audouin and Milne-Edwards, as heterosexual pairs (Pope 1946; Gray 1961; Haig 1965; Ng and Sasekumar 1993; Osawa 2001, 2007; Sanford 2006; Osawa and Poupin 2013; Britayev et al. 2017). In some cases commensalism seems to be facultative, as free-living individuals have been sporadically found in shallow waters under stones or in sand (Haig 1956, 1964; Werding 2001).

The *Polyonyxbiunguiculatus* group contains six species, four distributed in the Central Pacific (Osawa 2015) and two in the Indian Ocean. The Central Pacific species, *Polyonyxbiunguiculatus*, *P.obesulus*Miers 1884, *P.similis*Osawa 2015, and *P.triunguiculatus*Zehntner 1894, do not seem to have commensal relationships with other invertebrates, although they seem to prefer habitats characterized by corals and sponges (Haig 1964, 1979; Osawa 2007, 2015). The two Indian Ocean species, *P.hendersoni*Southwell 1909, and *P.splendidus*Sankolli 1963, have been rarely found, probably because they inhabit the ducts of sponges. Such life habit is reflected in the distinctive morphology of these two species (see Hiller et al. 2010), which made Werding (2001) consider them as conspecifics of a new genus. However, unpublished molecular data indicate that these species are aberrant forms of the *P.biunguiculatus* group.

In a recent study of symbionts of two syntopic species of chaetopterid polychaetes in the South China Sea of Vietnam, Britayev et al. (2017) found heterosexual pairs of a small porcellanid inhabiting one of this chaetopterid species. This porcellanid is an undescribed species of the *Polyonyxsinensis* group, which we here describe as *Polyonyxsocialis* sp. n. The new species was reported by Britayev et al. (2017) to share its host either with *P.heok* Osawa & Ng, 2016, a rather large porcellanid, and a nudibranch species of the genus *Phestilla* Bergh, or with a male-female pair of a pinnotherid crab of the genus *Tetrias* Rathbun.

## Materials and methods

Material of *Polyonyxsocialis* sp. n. was provided by T Britayev (Severtzov Institute of Ecology and Evolution, Russian Academy of Sciences, Moscow, Russian Federation) and D Martin (Department of Marine Ecology, Centre d’Estudis Avançats de Blanes, Blanes, Catalunya, Spain), and has been deposited in the Naturmuseum Senckenberg (SMF), Frankfurt a.M., Germany. Colour photographs were provided by T Britayev, and are included in the description. Measurements of carapace length and width (in mm) of type individuals follow collection information.

## Systematics

### Family Porcellanidae

#### Polyonyx socialis sp. n.

Taxon classificationAnimaliaDecapodaPorcellanidae

http://zoobank.org/C8712225-9D4B-40AA-87CA-0B6FCDB3174A

Figs 1 , 2a–g , 3

##### Material.

**Figure 1.**
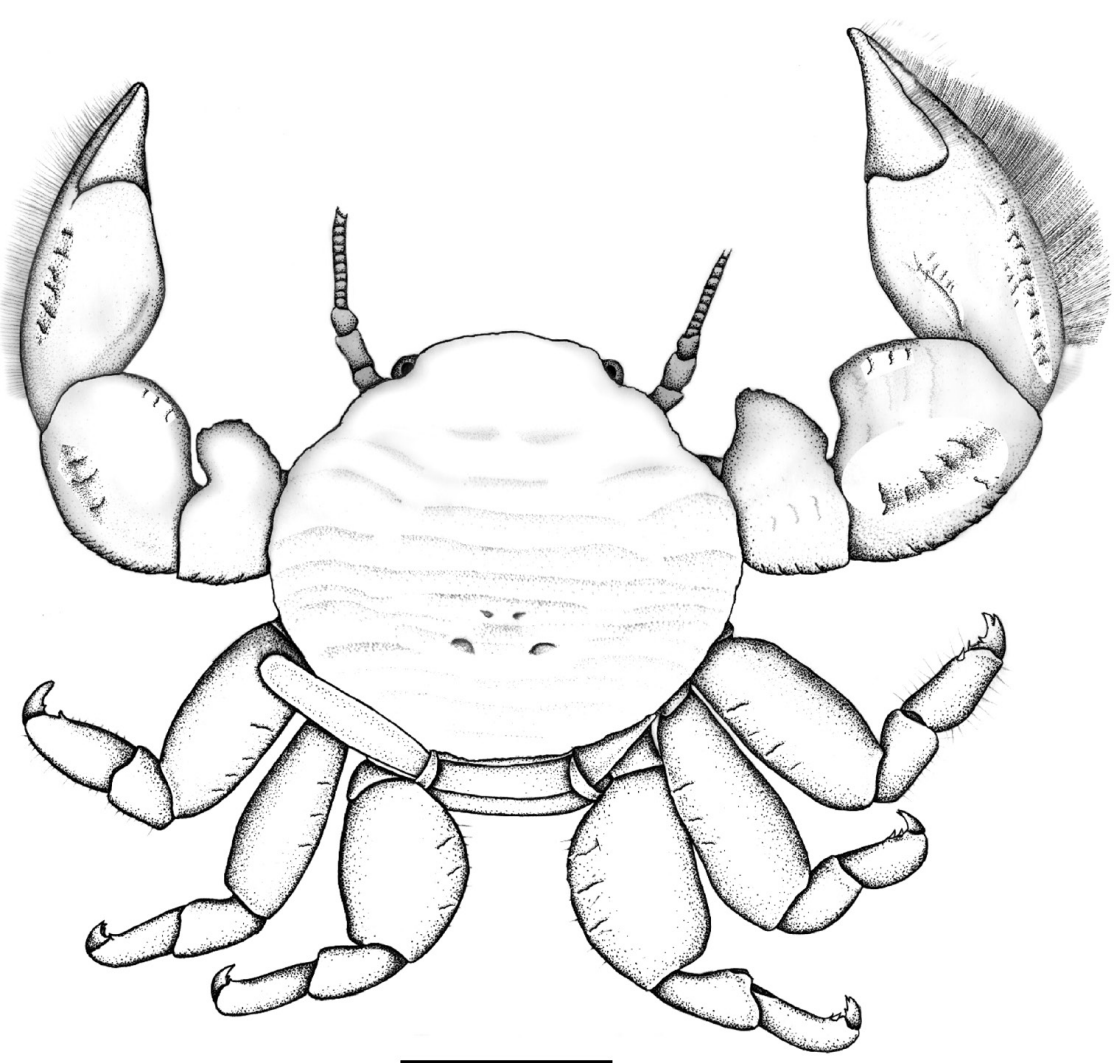
*Polyonyxsocialis* sp. n., female holotype, dorsal view, SMF 52400, South China Sea, south coast of Vietnam, Nhatrang Bay, Tre Island, Dam Bay, dorsal view. Scale bar: 2.0 mm.

**Figure 2.**
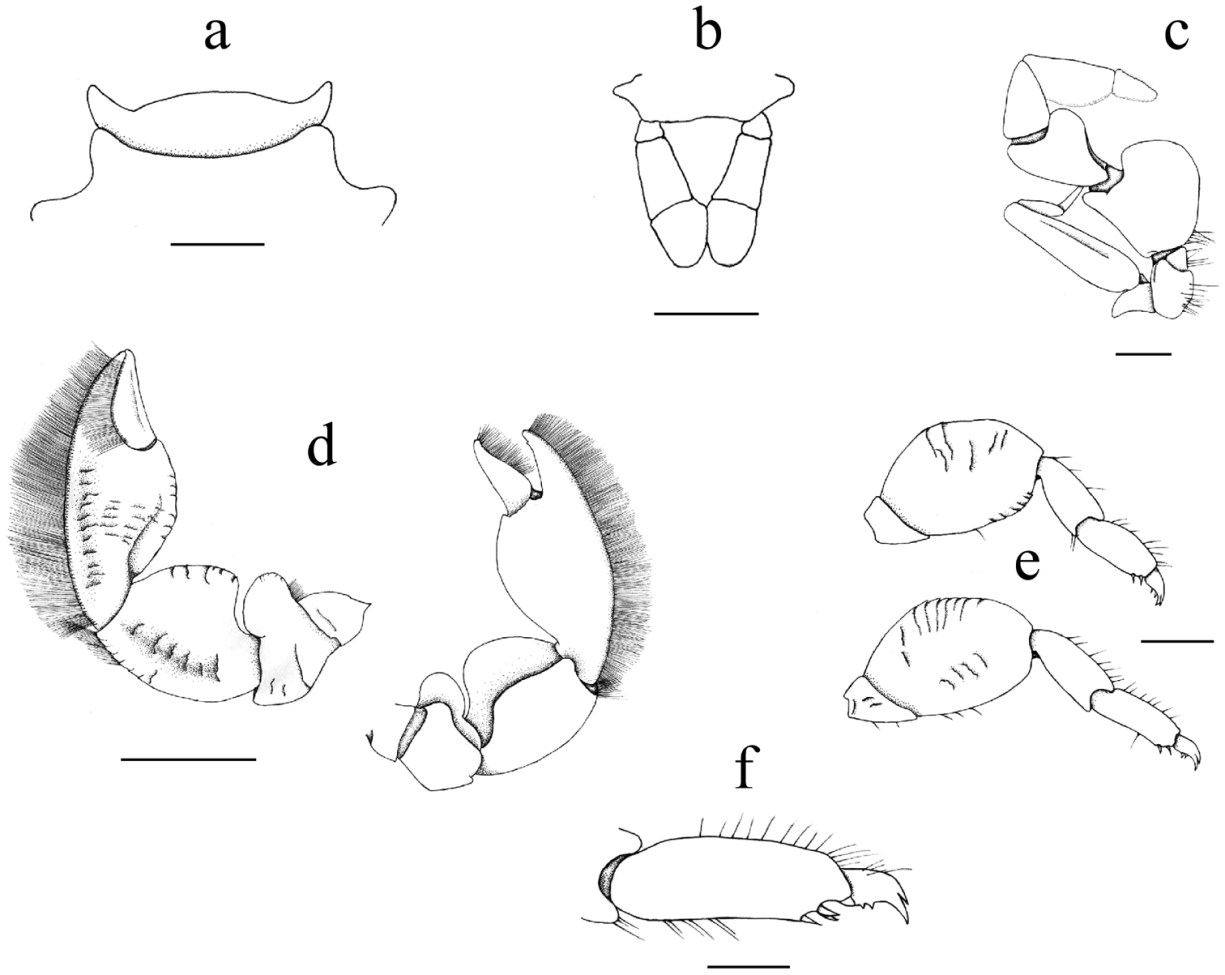
*Polyonyxsocialis* sp. n., male paratype, SMF 52401, South China Sea, south coast of Vietnam, Nhatrang Bay, Mun Island. **a** Third thoracic sternite **b** Telson **c** Right third maxilliped, dorsal view (setae omitted) **d** Left (larger) cheliped, dorsal and ventral views **e** First and second right walking legs, dorsal view **f** Detail of dorsal view of propodus and dactylus of right third walking leg. Scale bars: 0.5 mm (**a–c, f**), 2.0 mm (**d**), 1 mm (**e**).

**Figure 3.**
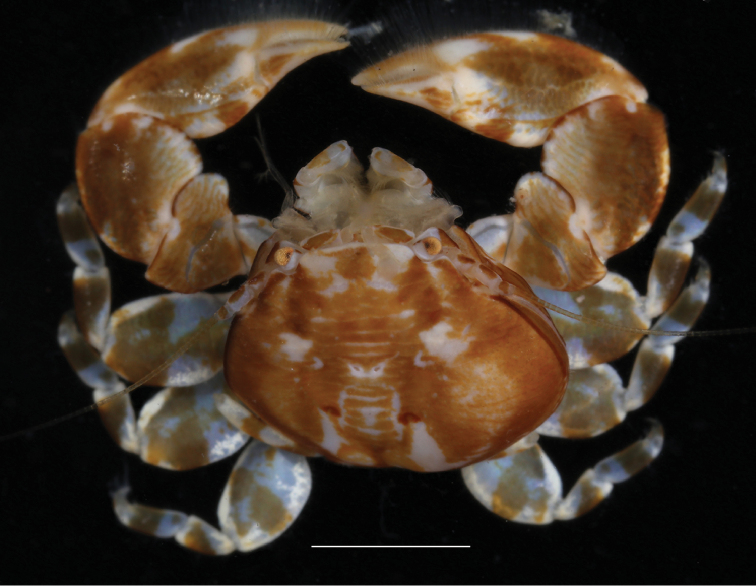
*Polyonyxsocialis* sp. n., female paratype, SMF 52401, South China Sea, south coast of Vietnam, Nhatrang Bay, Mun Island. Right side of carapace deformed by parasitic isopods (Bopyridae). Scale bar: 3.0 mm.

**Holotype**: female, SMF 52400, South China Sea, south coast of Vietnam, Nhatrang Bay, Tre Island, Dam Bay, 6–8 m, silty sand, hand collection from tube of *Chaetopterus* sp. No 66, cohabiting with a pair of pinnotherid *Tetrias* sp., coll. Britayev and Martin, 15.04. 2016; 4.0 mm x 4.3 mm (Fig. 1). **Paratypes**: male-female pair cohabiting with a pair of *Polyonyxheok*, SMF -52401, South China Sea, south coast of Vietnam, Nhatrang Bay, Mun Island, 16–20 m, silty sand, hand collection from tube of *Chaetopterus* sp. No 4, coll. Britayev and Martin, 04. 2016; male 4.6 mm x 5.7 mm (Fig. 2), female (ov) 5.1 mm x 5.6 mm (Fig. 3), both with bopyrid infestation and therefore, largely deformed.

##### Description.

Carapace (Fig. 1) round to subovate, 1.1 to 1.2 times wider than long, broadest at epibranchial level, moderately convex, surface smooth, shining, covered with distant, shallow transversal striae. Regions scarcely demarcated. Hepatic margin roundly produced, crested. Branchial margins evenly rounded, crested. Rostrum broad, transverse (holotype) or weakly trilobate (paratypes), median lobe forwardly directed, lateral lobes rounded. Orbits shallow, outer orbital angles rounded. Side walls entire, not visible from above.

Third thoracic sternite (Fig. 2a) broad, anterior margin rounded, lateral lobes broad, forwardly directed.

Telson composed of seven plates (Fig. 2b).

Basal article of antennular peduncle unarmed. First antennal article broadly in contact with lower orbital margin, movable articles smooth, second elongate; flagellum long, reaching to tip of chelae.

Third maxilliped (Fig. 2c) with broad ischium and rounded inner projection; merus with subrectangular rounded inner lobe. Exopod long, slender, overreaching middle of merus.

Chelipeds (Figs 1, 2d) similar in both sexes, heterochaely not very pronounced. Merus with some transverse rugae on upper surface, with large, laminate, forwardly projected lobe; carpus swollen, with similar lobe that makes carpus appear nearly as broad as long; upper surface with some transversal rugae, proximal border concave, outer border with faint, scale-like rugae, and scattered, short setae; distal portion with tuft of simple setae. Manus compact, swollen above, outer border evenly curved outside. Fingers short, approximately 1/3 of total length of manus. Outer border with narrow fringe of densely set, very fine, simple setae. Fingers closing on entire length, movable finger with upper border of cutting edge with fringe of upwardly standing simple setae.

Walking legs (Figs 2e–f) relatively short, merus flattened, ovate from above, 1.4 to 1.5 times longer than wide, surface with scattered, transversal ridges and scattered setae. Carpus and propodus elongate, of similar length, with scattered simple setae, propodus spineless except for terminal triplet. Dactylus elongate, terminating in curved, bifurcate claws, the upper one being smaller, inner margin with two or sometimes three smaller spines.

Males with pair of pleopods on second abdominal segment.

The overall coloration of *Polyonyxsocialis* sp. n. (Fig. 3) is light brown with a symmetric pattern of white marks on the carapace. The chelipeds have whitish marks upon the articulation between merus and carpus, and at the level of the articulation with the dactylus. The walking legs have white areas on the proximal part of the merus, and around the articulations.

##### Ecology.

*Polyonyxsocialis* sp. n. inhabits the tubes of a *Chaetopterus* sp. worm as heterosexual pairs, and shares its host with other symbionts, either a male-female pair of the porcellanid *P.heok* and the aeolid nudibranch *Phestilla* sp., or a male-female pair of the pinnotherid crab *Tetrias* sp. The hosts were collected between 6 and 20 m depth.

##### Etymology.

The specific name *socialis*, from the Latin, meaning social, refers to the sociable behaviour of the new species, as it tolerates and is tolerated by other symbionts inhabiting the same polychaete host.

##### Remarks.

The new species is morphologically similar and probably systematically close to *P.utinomii* Miyake, 1943 and *P.boucheti* Osawa, 2007, both of which also live in *Chaetopterus* tubes (Osawa 2001). The new species is distinguished from *P.utinomii* and *P.boucheti* by 1) the narrower carapace, with the rostrum being less transverse, 2) the extremely extended and forwardly directed lobes on merus and carpus of the chelipeds, 3) the wide and flattened merus of the walking legs, and 4) the extremely fine and transparent fringes of setae on the chelipeds.

##### Distribution.

Currently known only from the Vietnamese coast of the South China Sea.

## Discussion

*Polyonyxsocialis* sp. n. inhabits as a heterosexual pair the tubes of one of two syntopic species of *Chaetopterus*, which according to Britayev et al. (2017), may be a new undescribed species of polychaete from the Vietnamese South China Sea. These authors reported the new porcellanid frequently sharing the polychaete tube with a heterosexual pair of the significantly larger porcellanid *P.heok* and of the tergipedid nudibranch *Phestilla* sp. In one case *Polyonyxsocialis* sp. n. shared its host with a male-female pair of the pinnotherid crab *Tetrias* sp. Interestingly, *P.socialis* sp. n. was not found in the tubes of the larger polychaete Chaetopteruscf.appendiculatus Grube, 1874, which is ecologically close to the porcellanid’s host. The inhabitants of the larger polychaete were either the porcellanid *Eulenaioscometes* (Walker, 1887) and the polynoid polychaete *Ophthalmonoepettiboneae* Petersen & Britayev, 1997, or the carapid fish *Onuxodonfowleri* (Smith 1955). The presence of *P.socialis* sp. n. in the smaller and not the larger polychaete species may be explained by the crab’s host specificity, or by a lower tolerance of Chaetopteruscf.appendiculatus and its commensal inhabitants to sharing the space inside the tube.

The extremely broadened chelipeds and walking legs of *P.socialis* sp. n. are distinctive characters within *Polyonyx*, even when comparing the species with the morphologically closest *P.boucheti* and *P.utinomii*, and to all other tube-dwelling species of the genus. These characters are most likely adaptations to living tightly attached to the walls of the worm tube without being perceived as an obstacle for the larger crabs inhabiting the same tube. The new species is therefore morphologically adapted to cohabiting with a heterosexual pair of a congeneric larger crab, what is quite exceptional. The West Atlantic representative of the *P.sinensis* group, *P.gibbesi* Haig, 1956 inhabits the polychaete host *Chaetopterusvariopedatus* (Renier 1804) as a male-female pair (Gray 1961), and very rarely shares its host with the pinnotherid crab *Pinnixachaetopterana* Stimpson, 1860, which also inhabits the polychaete tube as a heterosexual pair (Grove and Woodin 1996). Studies in North Carolina and in the northern Gulf of Mexico (Pearse 1913, Gray 1961, Williams 1984, McDermott 2005) revealed that these two crab species do not coexist in one worm tube. In seldom cases, only juveniles of the two species were found in one tube. Apparently, the first to colonize the tube impedes the arrival of the other species. Once a heterosexual pair of either crab species has been established in the tube, it transitionally tolerates or completely rejects adults of the other species, which suggests that occupancy of the host by adult crabs involves intra- and interspecific competition (Sanford 2006). The cases where only one crab individual is present in the worm tube are probably transitional situations towards forming a male-female pair.

*Polyonyxsocialis* sp. n. and *P.heok* comprise the first pair of porcellanid crabs observed to share the same host.

## Supplementary Material

XML Treatment for Polyonyx socialis
